# Catastrophic total costs in tuberculosis-affected households and their determinants since Indonesia’s implementation of universal health coverage

**DOI:** 10.1186/s40249-017-0382-3

**Published:** 2018-01-12

**Authors:** Ahmad Fuady, Tanja A. J. Houweling, Muchtaruddin Mansyur, Jan Hendrik Richardus

**Affiliations:** 1 Department of Public Health, Erasmus MC, University Medical Center Rotterdam, P.O. Box 2040, 3000CA Rotterdam, The Netherlands; 2 Department of Community Medicine, Faculty of Medicine, Universitas Indonesia, Jakarta, Indonesia

**Keywords:** Tuberculosis, Catastrophic total cost, Determinant, Indonesia

## Abstract

**Background:**

As well as imposing an economic burden on affected households, the high costs related to tuberculosis (TB) can create access and adherence barriers. This highlights the particular urgency of achieving one of the End TB Strategy’s targets: that no TB-affected households have to face catastrophic costs by 2020. In Indonesia, as elsewhere, there is also an emerging need to provide social protection by implementing universal health coverage (UHC). We therefore assessed the incidence of catastrophic total costs due to TB, and their determinants since the implementation of UHC.

**Methods:**

We interviewed adult TB and multidrug-resistant TB (MDR-TB) patients in urban, suburban and rural areas of Indonesia who had been treated for at least one month or had finished treatment no more than one month earlier. Following the WHO recommendation, we assessed the incidence of catastrophic total costs due to TB. We also analyzed the sensitivity of incidence relative to several thresholds, and measured differences between poor and non-poor households in the incidence of catastrophic costs. Generalized linear mixed-model analysis was used to identify determinants of the catastrophic total costs.

**Results:**

We analyzed 282 TB and 64 MDR-TB patients. For TB-related services, the median (interquartile range) of total costs incurred by households was 133 USD (55–576); for MDR-TB-related services, it was 2804 USD (1008–4325). The incidence of catastrophic total costs in all TB-affected households was 36% (43% in poor households and 25% in non-poor households). For MDR-TB-affected households, the incidence was 83% (83% and 83%). In TB-affected households, the determinants of catastrophic total costs were poor households (adjusted odds ratio [a*OR*] = 3.7, 95% confidence interval [*CI*]: 1.7–7.8); being a breadwinner (a*OR* = 2.9, 95% *CI*: 1.3–6.6); job loss (a*OR* = 21.2; 95% *CI*: 8.3–53.9); and previous TB treatment (a*OR* = 2.9; 95% *CI*: 1.4–6.1). In MDR-TB-affected households, having an income-earning job before diagnosis was the only determinant of catastrophic total costs (a*OR* = 8.7; 95% *CI*: 1.8–41.7).

**Conclusions:**

Despite the implementation of UHC, TB-affected households still risk catastrophic total costs and further impoverishment. As well as ensuring access to healthcare, a cost-mitigation policy and additional financial protection should be provided to protect the poor and relieve income losses.

**Electronic supplementary material:**

The online version of this article (10.1186/s40249-017-0382-3) contains supplementary material, which is available to authorized users.

## Multilingual abstracts

Please see Additional file 1 for translations of the abstract into the five official working languages of the United Nations.

## Background

The estimated 1.4 million deaths to tuberculosis (TB) in 2015 exemplify the persisting burden of TB. With a global incidence that declines by only 2% annually worldwide, slow progress is being made towards the target for eliminating the disease by 2035 [1, 2]. These stark figures show that global action should be taken to adjust strategies and to combine initiatives such as promoting clinical adherence and providing socio-economic support [3, 4].

Although TB patients in most high TB-burden countries have free access to anti-TB drugs, they often incur high costs for travel and food, and suffer income losses that can amount to over half of annual household income [5, 6]. Such financial hardship creates an adherence barrier to diagnostic procedures and treatment, resulting in poor outcomes and increasing the risk of transmission in the community [5–8]. Accessing TB-related services also has economic consequences. The job or income losses experienced by TB patients, especially those in the productive age group, can reduce the financial capacity of their households and cast them into the poverty trap [9–11].

To address the socio-economic determinants and financial impact of TB, the WHO End TB Strategy acknowledges the need for social protection by setting a clear first milestone that no TB-affected families should face catastrophic TB-related costs after 2020 [1, 2]. This target complements the Sustainable Development Goal (SDG) of achieving universal health coverage (UHC) through the provision of more affordable and high-quality healthcare services [3, 12].

Indonesia started its UHC program in 2014 by offering national public insurance and by engaging more private providers in the network managed by the Social Security Agency (*Badan Penyelenggara Jaminan Sosial*, BPJS), the Ministry of Health and the Ministry of Social Affairs. It is assumed that direct medical costs, which are costs incurred for diagnostic tests, treatment, and follow-up tests, will be reduced by the national insurance scheme, which covers all medical costs in primary to tertiary care, including TB-related services [13]. Due to Indonesians people’s strong preference for seeking care with private providers, the involvement of more private providers in the BPJS network is also expected to have an impact by reducing medical expenses which were reportedly three times higher than those charged by public providers [14], and by reducing the number of people who develop TB but are not diagnosed or cannot access TB care services that conform with International Standard of Tuberculosis Care (ISTC) [9].

Accessing healthcare services is time-consuming and costly [9, 10, 15–17]. The Indonesian National TB Program (NTP) has attempted to provide support in the form of food/nutritional supplementation and travel vouchers, for example, in addition to diagnostic examination and drug costs coverage. However, the policy has changed and the support has been restricted or even ended. It leaves direct non-medical costs including travel and food/nutritional supplement costs uncovered and can lead to catastrophic health expenditure (CHE). As TB and multidrug-resistant TB (MDR-TB) require a long period of treatment, and also worsen health status, TB patients also suffer from job or income losses that aggravate the risk of catastrophic costs and barriers to treatment adherence.

The WHO has introduced a new term “catastrophic total costs” as the TB-specific indicator that differs in essence from CHE. CHE is defined as the share of the population spending more than a given threshold and focuses on direct cash spending or out-of-pocket (OOP) payments made by household to improve or restore health of household members. The TB-specific indicator of “catastrophic total costs” incorporates direct medical costs, direct non-medical costs and overall indirect costs, and helps to capture the economic burden specific for TB [18, 19]. It is therefore crucial for TB elimination programs to identify the main cost drivers, monitor financial hardship, and establish which further health and social policy measures should be taken [18]. For this reason, we aimed not only to measure the incidence of catastrophic total costs caused by TB and the sensitivity of the incidence relative to a range of specific thresholds, but also to assess differences between poor and non-poor households in terms of the incidence of catastrophic total costs and to identify the determinants of catastrophic total costs since Indonesia’s implementation of UHC.

## Methods

### Study design

From July to September 2016, a stratified clustered sampling design was used to enroll TB patients in an urban district (Jakarta), a suburban district (Depok) and a rural district (Tasikmalaya). Per district, we randomly selected 6–8 primary health centers (PHCs) linked with the NTP. Until reaching our predetermined sample size, we enrolled all the consecutive TB patients who attended these PHCs and who also met our inclusion criteria: they were aged 18 years or above, had undergone the adult diagnostic procedure, had been treated for at least one month or had finished treatment no more than a month previously, and had signed informed consent. Extra-pulmonary TB cases were excluded. Assuming a power of 0.80, a 1:1:1 ratio of urban to suburban to rural districts, and that the incidence of TB-related catastrophic total costs in each district was 20%, 25%, and 30%, we collected a minimum of 90 patients in each district.

MDR-TB patients were enrolled at Persahabatan Hospital, an MDR-TB referral hospital in Jakarta. We selected those adult MDR-TB patients who came to the hospital consecutively, had undergone MDR-TB treatment in the hospital for at least one month, had recorded a diagnostic result as MDR-TB, either by GenXpert or sputum culture; and had signed the informed consent form.

### Cost measurement

Ten medical students and public health graduates were recruited and trained as interviewers. Using the adapted Bahasa Indonesia version of the Tool to Estimate Patient Costs, they then interviewed patients and/or their drug observer, i.e., a family member who was selected as the patient’s direct-observation-of-treatment supporter [20, 21]. Retrospectively, each respondent reported all types of cost related to the TB care services they had incurred during the pre-diagnostic, diagnostic, and treatment phases (Table 1).

**Table 1:** Definition of costs and income used in this study

Variables	Definition	Direct costs	Indirect costs
Pre-diagnostic and diagnostic costs	All types of cost incurred during the period between the onset of symptoms and diagnosis with TB in public or private healthcare facilities, at a pharmacy, or by a practitioner of alternative medicine.	*Medical*: Costs of consultation, administration, laboratory tests, X-ray examinations, and drugs. *Non-medical*: Costs of food and travel for patient and/or guardian.	Patient’s and guardian’s income losses.
Treatment costs	All types of cost incurred after being diagnosed and treated for TB, includes the costs of hospitalization and adverse events.	*Medical*: Costs of administration, evaluation (laboratory test, X-ray examination, or others), hospitalization, and adverse events. *Non-medical*: Costs of food and travel (for patient and/or guardian), and food supplements.	Patient’s and guardian’s income losses.

#### Pre-diagnostic and diagnostic costs

The pre-diagnostic and diagnostic costs were the sum of all the direct and indirect costs incurred for pre-diagnostic and diagnostic visits. The direct costs included all OOP payments incurred after any reimbursement for medical fees and all non-medical expenditures made by patients or their guardian (i.e., a family member who accompanied them during visits). Indirect costs consisted of the income loss reported by patients and guardians.

#### Treatment costs

The costs of anti-TB drugs are covered by the NTP. We calculated the administration or registration fee, food and travel costs that were typical for each visit. To estimate the costs per month, we then multiplied these cost items by the number of visits per month. Any travel vouchers given to patients were included as a deduction of travel costs. We also summed treatment evaluation costs according to the number of evaluation tests conducted. We estimated patient’s income losses on the basis of income changes reported after diagnosis. To avoid underestimates for people such as taxibike drivers who continued to earn uncertain monthly incomes from informal jobs, we also estimated time-loss value. To calculate this time-loss value, we used the following formula: round trip in minutes for a typical visit × patient’s income loss per minute × the number of visits per month [5].

We interviewed some patients in the intensive treatment phase and others in the continuation treatment phase. For patients interviewed during the intensive phase, we obtained the reported costs of the intensive phase from the patient and estimated the costs in the continuation phase on the basis of other patients’ data in other PHCs within a same district. For patients interviewed during the continuation phase, we obtained reported costs from the patient in both the intensive and continuation phases, then extrapolated the reported costs to obtain the total costs of both phases. To estimate the entire treatment costs, we extrapolated the monthly costs according to the internationally defined durations of the intensive and continuation phases: (a) two months (for the intensive phase) and four months (for the continuation phase) of new TB treatment (Category I); (b) three and five months for re-treatment (Category II), and (c) eight and twelve months for MDR-TB treatment [5, 22, 23].

We summed other direct medical costs, e.g. hospitalization and any adverse event costs, that were uncovered by health insurance. We also calculated monthly nutritional/food supplement costs incurred by patients, such as vitamins, fruit, milk, meat, or other supplements consumed as a result of TB treatment.

To measure income loss, we established the household income earned through the incomes of patients and other family members, through government aid, and through other income, before and after the patients had been diagnosed with TB. A household earning below 1.9 USD per capita per day was classified as a poor household [24]. As many Indonesians live in extended families that may have more than one income earner per household, we defined a patient as breadwinner if his/her income was at least 10% higher than that of any other family member [25, 26]. All costs and incomes were converted to US dollars using the average exchange rate calculated by the World Bank for 2015 (1 USD = 13 389.41 IDR) [27].

### Catastrophic total costs

The WHO protocol takes two approaches to measure the percentage of patients experiencing catastrophic total costs. The first is based on total costs, and defines catastrophic total costs as total costs (direct and indirect costs) incurred by household that exceed 20% of the household’s annual income. The second approach defines catastrophic total costs as the share of TB patients who experience dissaving by taking a loan or selling property or livestock to deal with costs related to TB [18]. In this study, we applied the first approach. Total costs due to TB were defined as the sum of the OOPs incurred for medical diagnosis and treatment (OOPM), OOPs for non-medical expenditures related to the use of TB care services (OOPNM), and patients’ and guardians’ reported income losses or time losses valuations (IN), net of any reimbursement and welfare payments. The denominator was reported annual household income in the year before diagnosis with TB [18].

$$ I{\displaystyle \begin{array}{c} TB\\ {} NTP\end{array}}=\frac{1}{n\begin{array}{c} TB\\ {} NTP\end{array}}\sum \limits_{i=1}^{n\ \begin{array}{c} TB\\ {} NTP\end{array}}1\left(\sum \begin{array}{c}{n}_i\\ {}j=1\end{array}\frac{OOPM\begin{array}{c} TB,h\\ {}j\end{array}+ OOPNM\ \begin{array}{c} TB,h\\ {}j\end{array}+ IN\ \begin{array}{c} TB,h\\ {}j\end{array}}{y\begin{array}{c}h\\ {}i\end{array}}>{\tau}^{TB}\right) $$

As well as measuring the incidence of catastrophic total costs, referred to here as the headcount (H), we established the sensitivity of this headcount (i.e. incidence) relative to thresholds of 5%, 10%, 15%, 20%, and 25% as used in other previous studies [10, 11]. For each threshold, we also calculated mean gap (G) and mean positive gap (MPG). The G indicates the average amount by which payments, as a proportion of household income, exceed the threshold. The MPG is equal to G/H, and helps to identify how excessive the total costs are by indicating the excess expenditure per household that experiences catastrophic total costs [7, 11, 28, 29].

To analyze the different pictures provided by the catastrophic total cost approach and the CHE approach, we compared the H’s, G’s, and MPGs per threshold between these two approaches [30, 31]. Per threshold, we also analyzed differences between poor and non-poor households in the H’s, G’s, and MPGs of catastrophic total costs.

Fourteen patient variables were examined as potential determinants of catastrophic total costs: (1) district (urban, suburban, rural), (2) household income (poor and non-poor), (3) sex, (4) age group, (5) educational level (primary school as “low,” junior school and senior high school as “intermediate”; and college and university as “high”), (6) being a family breadwinner, (7) having had an income-earning job before diagnosis, (8) having insurance before being diagnosed, (9) having had previous TB treatment, (10) HIV status, (11) hospitalization for the current TB treatment, (12) first contact with the facility after having symptoms of TB, (13) taking Food supplementation, and (14) experiencing adverse effects.

### Data analysis

To ensure data quality, we used Microsoft Excel 2010 and EpiInfo version 7 (CDC, Atlanta) to double-enter and to check the data. Abnormally distributed data were displayed as median (inter-quartile range [IQR, q25-q75]), while categorical variables were shown as numbers and proportions (%). The Mann-Whitney test was used to compare all types of the cost incurred for access TB-related services between poor and non-poor households.

We used random effects to adjust for our cluster sampling design (19 PHCs), and used the generalized linear mixed model to examine determinants of the incidence with which TB-affected households faced catastrophic total costs. For MDR-TB cases, we used binary logistic regression to examine the determinants of catastrophic total costs. In the univariate analysis, we estimated the significance (*P*), the crude odds ratios (c*OR*s), and their 95% confidence intervals (*CI*s). To identify the best model and estimate the significances, adjusted *OR*s (a*OR*s) and the 95% *CI*s of the determinants, we included all variables with a *P* < 0.25 in the univariate analysis in a multivariable analysis.

### Ethical issues

Before the interview, all respondents received written and oral explanations of the study and signed an informed-consent form. Ethical clearance for this study was provided by the Ethical Committee at the Faculty of Medicine of Universitas Indonesia–Cipto Mangunkusumo Hospital, Jakarta, Indonesia (No. 416/UN2.F1/ETIK/VI/2016) and the Ethical Committee at Persahabatan Hospital, Jakarta, Indonesia (No. DL.01.03/II.3/3817/2016).

## Results

### Patients characteristics

As eight (3%) of the 354 eligible TB and MDR-TB patients did not report their household income, we analyzed the data from 346 patients (282 TB and 64 MDR-TB patients) (Table 2). Most patients were of working age, had an intermediate educational background, and lived in a poor household. Thirty-two percent of the TB patients with an income-earning job had lost their job after diagnosis, against 69% of the MDR-TB patients. Less than one-third (23%) of the TB patients in the urban study area did not have health insurance, compared with 59% in the rural study area. Most patients had smear-positive TB and were divided equally according to the phase of treatment.

**Table 2:** Patient characteristics

Characteristics	TB (%)								MDR-TB (%)	
	Total		Urban		Suburban		Rural			
Sex	*n = 282*		*n = 95*		*n = 90*		*n = 97*		*n = 64*	
Male	155	(55)	51	(54)	52	(58)	52	(54)	31	(48)
Female	127	(45)	44	(46)	38	(42)	45	(46)	33	(52)
Age in years										
18–40	137	(49)	45	(47)	47	(52)	45	(46)	34	(53)
41–64	123	(44)	44	(46)	38	(42)	41	(42)	29	(45)
> 64	22	(8)	6	(6)	5	(6)	11	(11)	1	(2)
Educational level										
Low	99	(35)	25	(26)	18	(20)	56	(58)	12	(19)
Intermediate	172	(61)	67	(71)	65	(72)	40	(41)	42	(65)
High	11	(4)	3	(3)	7	(8)	1	(1)	10	(16)
Household income										
Poor	175	(62)	46	(48)	45	(50)	84	(87)	23	(36)
Non-poor	107	(38)	49	(52)	45	(50)	13	(13)	41	(64)
Breadwinner										
Patient	124	(44)	48	(51)	38	(42)	38	(39)	25	(39)
Not patient	158	(56)	47	(49)	52	(58)	59	(61)	39	(61)
Income-earning job										
Yes	201	(71)	73	(77)	61	(68)	67	(69)	49	(77)
No	81	(29)	22	(23)	29	(32)	30	(31)	15	(23)
Job loss										
Job loss	64	(23)	17	(18)	18	(20)	29	(30)	34	(53)
No job loss	218	(77)	78	(82)	72	(80)	68	(70)	30	(47)
Having health insurance										
Yes	176	(62)	73	(77)	63	(70)	40	(41)	56	(87)
No	106	(38)	22	(23)	27	(30)	57	(59)	8	(13)
Insurance type	*n = 176*		*n = 73*		*n = 63*		*n = 40*		*n = 56*	
BPJS, (paid by government^a^)	119	(68)	52	(71)	33	(52)	34	(85)	24	(43)
BPJS, (self-paid^b^)	53	(30)	19	(26)	28	(44)	6	(15)	32	(57)
Private insurance	4	(2)	2	(3)	2	(3)	0	(0)	0	(0)
Monthly income	*n = 201*		*n = 73*		*n = 61*		*n = 67*		*n = 49*	
Paid regularly	90	(45)	32	(44)	39	(64)	19	(28)	34	(69)
Uncertain	105	(52)	37	(51)	20	(33)	48	(72)	13	(27)
Others	6	(3)	4	(5)	2	(3)	0	(0)	2	(4)
	*n = 282*		*n = 95*		*n = 90*		*n = 97*		*n = 64*	
Type of TB										
Pulmonary, smear +	186	(66)	70	(74)	62	(69)	54	(56)	64	(100)
Pulmonary, smear -	80	(28)	23	(24)	24	(27)	33	(34)	0	(0)
Pulmonary, smear unknown	16	(6)	2	(2)	4	(4)	10	(10)	0	(0)
Therapy phase										
Intensive phase	134	(48)	38	(40)	51	(57)	45	(46)	37	(58)
Continuation phase	148	(52)	57	(60)	39	(43)	52	(54)	27	(42)
HIV status										
Positive	6	(2)	5	(5)	1	(1)	0	(0)	0	(0)
Negative	92	(33)	51	(54)	17	(19)	24	(25)	32	(50)
Not tested/unknown	184	(65)	39	(41)	72	(80)	73	(75)	32	(50)
Hospitalization										
Yes	39	(14)	11	(12)	13	(14)	15	(16)	34	(53)
No	243	(86)	84	(88)	77	(86)	82	(84)	30	(47)
Previous TB treatment										
Yes	58	(21)	29	(31)	20	(22)	9	(9)	56	(87)
No	224	(79)	66	(69)	70	(78)	88	(91)	8	(13)
Completed previous TB treatment	*n = 58*		*n = 29*		*n = 20*		*n = 8*		*n = 56*	
Yes	35	(61)	19	(65)	10	(50)	6	(75)	34	(61)
No	22	(39)	10	(35)	10	(50)	2	(25)	22	(39)
First contact										
Public hospital	127	(45)	51	(54)	40	(44)	36	(37)	29	(45)
Private hospital	94	(33)	26	(27)	26	(29)	42	(43)	2	(3)
Primary health center	32	(11)	11	(12)	10	(11)	11	(11)	25	(39)
Private clinic	20	(7)	4	(4)	13	(14)	3	(3)	8	(13)
Other facility	9	(3)	3	(3)	1	(1)	5	(5)	0	(0)

^a^Their national public insurance (BPJS) premiums were paid by the government; ^b^They paid national public insurance (BPJS) premium out of their pocket

In spite of the availability of primary care, most TB patients first sought care in a hospital (78%). Our results also show that a high proportion of TB patients went first to a private provider; even in rural areas, this figure was 46%. Investigation of the reasons for not choosing a public provider showed that the distance to the public facility was a prominent issue, as were personal preference and familiarity with a specific private facility (See Additional file 2).

### TB-related total costs

**Fig. 1:**
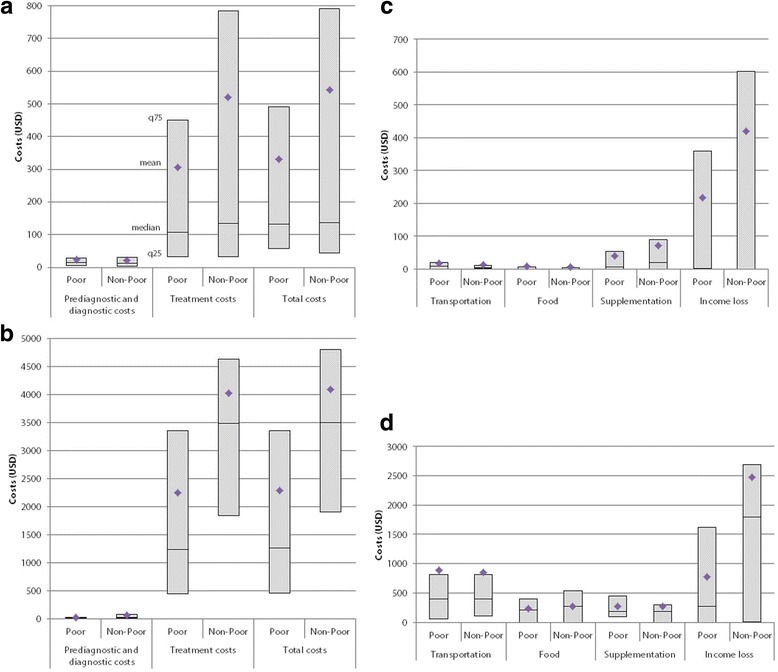
Costs incurred for TB-related services. Pre-diagnostic and diagnostic costs, treatment costs and total costs between poor and non-poor patients in (**a**) TB groups and (**b**) MDR-TB groups; and costs incurred during treatment in (**c**) TB and (**d**) MDR-TB affected households. Means are indicated by blue rhombs, medians by a horizontal line, q25 by the bottom horizontal line of each box, and q75 by the top horizontal line of each box

The median (IQR) of total costs was 133 USD in the TB group (55–576) and 2804 USD in the MDR-TB group (1008–4325) (Table 3). The treatment costs amounted to 88% of median total costs for TB patients and 98% for MDR-TB patients. (Figure 1) Despite the low medians of indirect costs and patients’ income loss, our results show that once patients lost their jobs, they lost a lot of their income. Among those who lost their jobs, average income loss amounted to 80% of total costs. Instead, the median annual income of TB and MDR-TB patients (1344 USD and 2241 USD) were much lower than the Indonesian GDP per capita in 2015 (3834 USD).

**Table 3:** Costs of pre-diagnosis, diagnosis, and treatment of TB stratified by income at household level; median (IQR) in USD

Costs	TB						*P*	MDR-TB						*P*
	Total		Poor		Non-Poor			Total		Poor		Non-Poor		
Prediagnosis and diagnosis														
Direct costs	11	(3–21)	12	(4–22)	10	(2–21)	0.857	21	(7–47)	15	(5–35)	28	(7–67)	0.164
Indirect costs	1	(0–7)	1	(0–7)	0	(0–4)	0.136	4	(0–16)	1	(0–8)	5	(0–19)	0.141
Patient’s income loss	0	(0–0)	0	(0–2)	0	(0–0)	0.089	0	(0–0)	0	(0–0)	0	(0–2)	0.428
Guardian’s income loss	0	(0–0)	0	(0–0)	0	(0–0)	0.393	0	(0–0)	0	(0–0)	0	(0–0)	0.650
Total	13	(5–30)	15	(5–29)	12	(3–31)	0.750	27	(13–62)	20	(11–35)	32	(14–85)	0.049
Treatment														
Direct medical costs	0	(0–6)	2	(0–6)	0	(0–3)	0.851	15	(0–78)	9	(0–60)	17	(0–116)	0.328
Administration costs	0	(0–2)	0	(0–3)	0	(0–2)	0.426	0	(0–0)	0	(0–0)	0	(0–0)	0.454
Treatment evaluation costs	0	(0–0)	0	(0–0)	0	(0–0)	0.104	0	(0–0)	0	(0–0)	0	(0–0)	0.188
Hospitalization costs	0	(0–0)	0	(0–0)	0	(0–0)	0.748	8	(0–68)	0	(0–60)	15	(0–78)	0.499
Adverse effect costs	0	(0–0)	0	(0–0)	0	(0–0)	0.739	0	(0–0)	0	(0–0)	0	(0–0)	0.869
Direct non-medical costs	33	(8–97)	29	(9–90)	40	(8–117)	0.666	856	(430–1427)	941	(430–1255)	807	(430–1546)	0.690
Travel costs	7	(1–17)	9	(0–21)	4	(1–10)	0.004	403	(108–807)	403	(54–807)	403	(108–807)	0.457
Food costs	0	(0–5)	0	(0–6)	0	(0–4)	0.660	269	(0–459)	215	(0–403)	269	(0–538)	0.442
Food supplement costs	13	(0–67)	7	(0–54)	20	(0–90)	0.641	179	(4–347)	179	(90–448)	179	(0–291)	0.416
Indirect costs	8	(0–448)	11	(0–358)	4	(0–602)	0.236	1344	(2–2577)	269	(0–1882)	1792	(202–2913)	0.009
Patient’s income loss	2	(0–448)	2	(0–358)	1	(0–602)	0.143	1344	(2–2577)	269	(0–1613)	1792	(2–2689)	0.015
Guardian’s income loss	0	(0–0)	0	(0–0)	0	(0–0)	0.380	0	(0–0)	0	(0–0)	0	(0–0)	0.465
Total	117	(33–545)	108	(33–451)	135	(32–784)	0.352	2760	(989–4309)	1244	(448–3352)	3485	(1851–4638)	0.012
Total costs	133	(55–576)	132	(58–492)	138	(45–792)	0.277	2804	(1008–4325)	1268	(461–3363)	3506	(1914–4799)	0.011

The differences in total costs between poor and non-poor TB patients were not statistically significant. However, in non-poor households affected by MDR-TB, the total costs were higher than in poor households, due mainly to higher income losses.

### Catastrophic total costs

At the 20% threshold, the incidence, i.e. headcount, of catastrophic total costs was 36% for TB and 83% for MDR-TB; this was similar to the respective incidences of CHE at the 10% threshold (22% and 84%) (Table 4). However, the catastrophic total costs approach consistently showed higher mean gaps (G’s) both for TB (10% vs. 4%) and MDR-TB (79% vs. 68%) than the CHE approach did.

**Table 4:** The headcounts of catastrophic costs due to TB and the sensitivity of these headcounts

Catastrophic costs	TB					MDR-TB				
	5%	10%	15%	20%	25%	5%	10%	15%	20%	25%
Catastrophic total costs^a^										
Headcount (%)		50	43	36	31		94	88	83	80
Mean gap (%)		14	12	10	8		88	83	79	75
Mean positive gap (%)		28	27	27	26		93	95	95	94
Catastrophic health expenditure^a^										
Headcount (%)	42	22	16	12		94	84	69	61	
Mean gap (%)	6	4	3	2		72	68	64	60	
Mean positive gap (%)	14	18	19	17		77	81	93	98	

^a^Catastrophic total costs approach incorporates all type of costs, i.e. direct medical costs, direct non-medical costs, and overall indirect costs, while the CHE approach focuses only on direct cash spending or OOP payments made by household

There was an inverse association between catastrophic total costs and household income. Although their median total costs were not significantly different, poor TB-affected households, which had lower incomes, had higher headcounts than non-poor households (43% vs. 25%, *P =* 0.006 when using the threshold of 20%) (Table 5). The differences in incidence of catastrophic total costs between poor and non-poor households were also statistically significant with the thresholds of 10% (*P* = 0.014), 15% (*P* = 0.006), and 25% (*P =* 0.009). For MDR-TB, the incidence of catastrophic total costs was similar for poor and non-poor households, irrespective to the threshold used. At the same time, the G’s indicated that poor households suffered more than non-poor households (138% vs. 45% when using threshold of 20%). As the MPGs indicated, the gap was greater in poor households that faced catastrophic total costs (167%).

**Table 5:** Differences between poor and non-poor households in catastrophic total costs

Catastrophic total costs	TB				MDR-TB			
	10%	15%	20%	25%	10%	15%	20%	25%
Headcount								
Poor (%)	57	50	43	37	96	87	83	78
Non-poor (%)	39	32	25	22	93	88	83	81
Total (%)	50	43	36	31	94	88	83	80
*P*	0.014	<0.006	0.006	0.009	0.111	0.117	0.115	0.121
Mean gap								
Poor (%)	18	15	13	11	147	142	138	134
Non-poor (%)	8	6	5	3	54	50	45	41
Total (%)	14	12	10	8	88	83	79	75
Meanpositive gap								
Poor (%)	32	30	30	30	154	164	167	171
Non-poor (%)	19	19	18	16	58	56	55	51
Total (%)	28	27	27	26	93	95	95	94

### Determinants of catastrophic total costs

With regard to catastrophic total costs among TB-affected households, there were four determinants: poor household (adjusted odds ratio [a*OR*] = 3.7; 95% confidence interval [*CI*]: 1.7–7.8; *P* = 0.001); breadwinners (a*OR* = 2.9; 95% *CI*: 1.3–6.6; *P* = 0.010); job loss (a*OR* = 21.2; 95% *CI*: 8.3–53.9; *P* < 0.001); and previous TB treatment (a*OR* = 2.9; 95% *CI*: 1.4–6.1; *P* = 0.006) (Table 6). Not being covered by health insurance was not a determinant of catastrophic total costs in either TB-affected or MDR-TB-affected households. With regard to MDR-TB-affected households, the multivariable analysis showed that the only determinant of catastrophic total costs in these households was having had an income-earning job before diagnosis (a*OR* = 8.7; 95% *CI*: 1.8–41.7; *P =* 0.007) (Table 7).

**Table 6:** Determinants of catastrophic total costs in TB cases

Determinants	Catastrophic total costs				*P*	c*OR* (95% *CI*)	*P*	a*OR* (95% *CI*)
	Yes	%	No	%				
Household income								
Poor	75	43	100	57	0.006	2.20 (1.26–3.86)	0.001	3.68 (1.74–7.78)
Non-poor	27	25	80	75		1.00		1.00
District								
Urban	35	37	60	63		1.00		
Sub-urban	22	24	68	76	0.125	0.54 (0.25–1.19)		
Rural	45	46	52	54	0.317	1.47 (0.69–3.16)		
Sex								
Male	57	37	98	63	0.710	1.10 (0.66–1.82)		
Female	45	35	82	65		1.00		
Age, years old								
18–40	49	36	88	64		1.00		
41–64	44	36	79	64	0.977	0.99 (0.59–2.67)		
> 64	9	41	13	59	0.816	1.12 (0.43–2.90)		
Educational level								
Low	41	41	58	59		1.00		
Intermediate	58	34	114	66	0.355	0.78 (0.45–1.33)		
High	3	27	8	73	0.479	0.60 (0.14–2.51)		
Breadwinner								
Patient	65	52	59	48	<0.001	3.60 (2.16–6.00)	0.010	2.92 (1.29–6.60)
Not patient	37	23	121	77		1.00		1.00
Income-earning job								
Yes	90	45	111	55	<0.001	4.66 (2.38–9.14)	0.881	1.08 (0.40–2.92)
No	12	15	69	85		1.00		1.00
Job loss								
Job loss	51	80	13	20	<0.001	14.07 (6.84–28.93)	<0.001	21.17 (8.31–53.90)
No job loss	51	23	167	77		1.00		1.00
Health insurance								
No	43	41	63	59	0.390	1.26 (0.74–2.15)		
Yes	59	34	117	66		1.00		
HIV status								
Negative	44	48	48	52		1.00		
Positive	0	0	6	100	0.953	0.00 (0.00-~)		
Not tested/unknown	58	32	126	68	0.863	0.46 (0.26–0.82)		
Previous TB treatment								
Yes	31	53	27	47	0.001	2.93 (1.56–5.48)	0.006	2.86 (1.35–6.05)
No	71	32	153	68		1.00		1.00
First contact with facility								
Private facility	46	37	77	63	0.622	1.14 (0.68–1.89)		
Public facility	56	35	103	65		1.00		
Hospitalization								
Yes	15	38	24	62	0.685	1.16 (0.56–2.38)		
No	87	36	156	64		1.00		
Food supplement								
Yes	65	34	126	66	0.370	0.78 (0.5–1.3)		
No	37	41	54	59		1.00		
Adverse effect								
Yes	53	43	71	57	0.029	1.77 (1.06–2.95)	0.089	1.77 (0.92–3.40)
No	49	31	109	69		1.00		1.00

c*OR* crude Odds Ratio, a*OR* adjusted Odds Ratio

**Table 7:** Determinants of catastrophic total costs in MDR-TB cases

Determinants	Catastrophic total costs				*P*	c*OR* (95% *CI*)	*P*	a*OR* (95% *CI*)
	Yes	%	No	%				
Household income								
Poor	19	83	4	17	0.974	0.98 (0.25–3.88)		
Non-poor	34	83	7	17		1.00		
Sex								
Male	27	87	4	13	0.383	1.82 (0.48–6.95)		
Female	26	79	7	21		1.00		
Age in years								
18–40	28	82	6	18		1.00		
>40	25	83	5	17	0.917	1.07 (0.29–3.95)		
Educational level								
Low	9	75	3	25		1.00		
Intermediate	35	83	7	17	0.515	1.67 (0.36–7.76)		
High	9	90	1	10	0.378	3.00 (0.26–34.58)		
Breadwinner								
Patient	21	84	4	16	0.840	1.15 (0.30–4.41)		
Not patient	32	82	7	18		1.00		
Income-earning job								
Yes	44	90	5	10	0.012	5.87 (1.47–23.47)	0.007	8.68 (1.81–41.70)
No	9	60	6	40		1.00		1.00
Job loss								
Job loss	34	100	0	0		1.00		
No job loss	19	63	11	37	0.998	0.00 (0.00-~)		
Health insurance								
Yes	46	82	10	18		1.00		
No	7	87	1	13	0.709	1.52 (0.17–13.79)		
HIV status								
HIV negative	28	87	4	13		1.00		
HIV not tested/unknown	25	78	7	22	0.331	0.51 (0.13–2.01)		
Previous TB treatment								
Yes	47	84	9	16	0.534	1.75 (0.29–2.01)		
No	6	75	2	25		1.00		
First contact facility								
Private facility	10	100	0	0	0.266	8.66 (0.19–403.74)		
Public facility	43	80	11	20		1.00		
Hospitalization								
Yes	30	88	4	12	0.235	2.27 (0.58–8.91)	0.090	3.92 (0.81–19.01)
No	23	77	7	23		1.00		1.00
Food supplement								
Yes	41	84	8	16	0.741	1.28 (0.29–5.77)		
No	12	80	3	20		1.00		
Adverse effect								
Yes	38	86	6	14	0.270	0.47 (0.13–1.79)		
No	15	75	5	25		1.00		

*cOR* crude Odds Ratio, *aOR* adjusted Odds Ratio

## Discussion

Despite the implementation of UHC in Indonesia, there is a high incidence of catastrophic total costs due to TB, particularly among patients who live in poor households and those who lose their jobs due to TB. In general, the greatest contribution to total costs was made by travel and food/nutritional supplementation costs. However, losing both job and income after diagnosis was also a critical point: once patients had lost their jobs, income loss became the main driver of total costs. These findings emphasize the importance not only of providing travel and nutritional supports but also social protection for those who lose income due to TB.

Unlike CHE, the catastrophic total costs approach which incorporates direct medical costs, direct non-medical costs, and overall indirect costs highlights the impact of income loss. It also provides a clearer description of the severity of the financial impact than the CHE approach does. This is indicated by the consistently higher mean gap in the TB and MDR-TB groups.

The determinants of catastrophic total costs shown in this study highlight both the magnitude of the problem of income loss and the need to address it properly. As well as aggravating barriers to TB treatment adherence, thereby potentially worsening TB outcomes, income loss increases the risk of catastrophic costs and even greater impoverishment. If a TB patient is the family breadwinner, the incidence of catastrophic total costs is doubled.

In MDR-TB patients, coming from a poor household was not a determinant of catastrophic total costs. We had assumed that most MDR-TB cases would come from poor households, but this proportion was in fact very low. Overall, the incidence of catastrophic total costs was also very high: irrespective of their income level, over half of MDR-TB-affected households experienced such costs.

As our findings provide insights that contrast with the perspective of CHE, they provide a new basis for estimating costs, and may thus have policy implications. As well as supporting the WHO’s recommendation that the catastrophic total costs approach should be used, the main implication of our study is a strong recommendation to government that it should introduce a cost-mitigation policy and additional social protection beyond free medical services [5, 17]. Forms of financial protection other than food/nutritional supplementation and travel vouchers may be required. Cash transfers could be made conditional on behavioral requirements such as continuing treatment. Microfinance programs are also a potential form of financial support [32, 33], but this strategy requires complex and expensive inputs. The government should target beneficiaries carefully, ensure the delivery to patients, provide incentives that induce patients to adhere to treatment, and should therefore consider reserving a budget that is large enough. As well as emphasizing financial incentives, it is imperative to tackle any stigma and discrimination in workplaces that can lead to income loss. The government should also strengthen the policy by supporting job protection or paid sick leave for formally employed TB patients.

The high incidence of catastrophic total costs among poor patients requires inputs within the UHC framework. The government should incorporate strategies for widening population coverage, for improving the availability, accessibility, and quality of public health facilities; and also for involving as many private health facilities as possible in the BPJS network. To conform with the ISTC, they should also ensure proper training.

This study has several limitations. First, in line with the WHO protocol, we collected data from TB patients who visited PHCs and excluded those who were treated in facilities that were not linked to the NTP. Neither did we include TB nor suspected TB patients who were unable to afford TB-related services or who dropped out of the diagnostic procedure or out of treatment. This may have led to an underestimation of the incidence of catastrophic total costs. Second, MDR-TB patients were only recruited in a pulmonary hospital in an urban area with a low proportion of poor households. We did not describe a situation in which patients were removed from the hospital to PHCs for taking MDR-TB drugs after sputum conversion, and dropped out from treatment. Third, although we interviewed patients with a structured questionnaire to help recall their spending, our findings may have been affected by recall biases. Finally, while our study results apply to the western part of Indonesia such as Java, Bali, and Sumatra, which constitute 80% of the Indonesian population [34], some parts of Indonesia may have different characteristics that require careful generalization, especially the islands and more remote areas.

## Conclusions

Both TB and MDR-TB patients are in danger of falling into even deeper poverty. Travel costs, food/nutritional supplementation costs, and income loss all contribute to the incidence of catastrophic total costs. This risk is higher in patients from poor households, especially when they are breadwinners who lose their jobs. These findings suggest that measures beyond free medical services are required to mitigate the financial burden of households affected by TB, particularly for patients living in at-risk groups.

## Additional files

Additional file 1: Multilingual abstracts in the five official working languages of the United Nations. (PDF 794 kb)

Additional file 2: Reasons for not choosing public facilities at the first contact. (DOCX 15 kb)

## Abbreviations

a*OR*: Adjusted odds ratio
BPJS: Badan Penyelenggara Jaminan Kesehatan
CHE: Catastrophic health expenditure
*CI*: Confidence interval
c*OR*: Crude odds ratio
G: Mean gap
H: Headcount
IQR: Interquartile range
ISTC: International Standard of Tuberculosis Care
MDR-TB: Multidrug-resistant tuberculosis
MPG: Mean positive gap
NTP: National Tuberculosis Program
OOP: Out-of-pocket
PHC: Primary Health Centre
TB: Tuberculosis
UHC: Universal health coverage
USD: US Dollar
WHO: World Health Organization
