# Supplementary material for: Catastrophic total costs in tuberculosis-affected households and their determinants since Indonesia’s implementation of universal health coverage
Source: Infect Dis Poverty. 2018 Jan 12;7:3. doi: 10.1186/s40249-017-0382-3 (PMC5765643; doi:10.1186/s40249-017-0382-3)
Supplement: Supplementary file 1 — Multilingual abstracts in the five official working languages of the United Nations. (PDF 794 kb) [file 40249_2017_382_MOESM1_ESM.pdf]

Translation of the abstract into the five official working languages of the United Nations

التكاليف الإجمالية الكارثية للأسر المتضررة من السل ومحدداتها منذ تنفيذ إندونيسيا للتغطية الصحية الشاملة

أحمد فوادي، طنجة أ.ج. هويلينغ ومختار الدين منصور وجان هندريك ريتشاردوس

الملخص

خلفية: وباإلضافة إلى فرض عبء اقتصادي على الأسر المتضررة، فإن التكاليف المرتفعة المرتبطة بالسل يمكن أن تنشئ حواجز للوصول والامتثال. و هذا يلقي الضوء على الطابع الملح الملحوظ لتحقيق أحد أهداف استراتيجية القضاء على السل: ألا تواجه الأسر المعيشية المتضررة من السل تكاليف كارثية بحلول عام 2020. وفي إندونيسيا حاجة ماسة إلى توفير الحماية الاجتماعية من خلال تنفيذ التغطية الصحية الشاملة، كما هو الحال في أماكن أخرى. ولقيمنا مجموع التكاليف بسبب السل ومحدداتها منذ تنفيذ UHC في حالات مأساوية.

الأساليب: <bx/>أجرينا المقابلة مع البالغين المصابين بالسل ومرضى السل (السل المقاوم للأدوية المتعددة) المقاوم للأدوية المتعددة في المناطق الحضرية والضواحي والمناطق الريفية في إندونيسيا الذين قد تمت معالجتهم لمدة شهر واحد على الأقل أو قد أنهي علاجهم قبل أقل من شهر. اتباعاً لتعليمات منظة الصحة العالمية، قمنا بتقييم مجموع التكاليف بسبب السل ومحدداتها في حالات مأساوية. نحن أيضا قمنا بتحليل الحساسية للإصابة بالنسبة إلى عتبات عدة، وقياس الاختلافات بين الأسر الفقيرة وغير الفقيرة في الإصابة بتكاليف كارثية. تحليل نموذج مختلط الخطي المعمم استخدمت لتحديد العوامل المحددة للتكاليف الإجمالية كارثية.

نتائج: <bx/>قمنا بتحليل السل 282 و 64 من مرضى السل المقاوم للأدوية المتعددة. الخدمات المتصلة بالسل، كان متوسط إجمالي التكاليف التي تتكبدها الأسر المعيشية (نطاق المجال) 133 دولاراً (55-576)؛ الخدمات المتعلقة بالسل المقاوم للأدوية المتعددة، كان الدولار 2 804 (1 008-4 325). وكان وقوع كارثة مجموع التكاليف في جميع الأسر المتأثرة بمرض السل 36% (43 في المائة في الأسر المعيشية الفقيرة ونسبة 25 في المائة في الأسر غير الفقيرة). للأسر المتضررة من السل المقاوم للأدوية المتعددة، كان معدل 83% (83% و 83%). كانت محددات التكاليف الإجمالية كارثية في الأسر المصابة بمرض السل، الأسر الفقيرة (تعديل نسبة الرجحان [أو] = 3.7، فاصل الثقة 95 ٪ [كايماني]: 1.7-7.8)؛ يجري عائل (أو= 2.9، CI%95: 1.3-6.6)؛ فقدان الوظيفة (أو= 21.2؛ CI%95: 8.3 53.9)؛ وعلاج السل السابقة (أو= 2.9؛ CI%95: 1.4-6.1). في الأسر المتضررة من السل المقاوم للأدوية المتعددة، بعد مهمة كسب الدخل قبل التشخيص المحدد فقط من التكاليف الإجمالية كارثية (أو= 8.7؛ CI%95: 1.8 41.7).

الاستنتاجات: <bx/>على الرغم من تنفيذ UHC، لا تزال الأسر المتضررة من السل معرضة لخطر مجموع التكاليف المفرطة وزيادة الفقر. فضلا عن ضمان إمكانية الوصول إلى الرعاية الصحية، ينبغي توفير سياسة التخفيف من التكاليف وحماية مالية إضافية لحماية الفقراء وتخفيف الخسائر في الدخل.

Translated from English version into Arabic by Mohammad Zahidul Islam, through

TRANSLATORS WITHOUT BORDERS

自印度尼西亚实施全民健康覆盖以来，受结核病影响家庭的灾难性总成本及其决定因素

Ahmad Fuady, Tanja A.J. Houweling, Muchtaruddin Mansyur, Jan Hendrik Richardus

摘要

引言：除了受影响家庭的经济负担之外，与结核病相关的高成本也会造成资源获取和依从性障碍。这凸显了实现消除结核病战略目标的紧迫性，即到 2020 年，受结核病影响的家庭无须面对

灾难性的成本负担。和其他地方一样，在印度尼西亚，可通过实施全民健康覆盖（UHC）来提供社会保障。因此，我们评估了结核病引起的灾难性总成本的发生率，以及自 UHC 实施以来的决定因素。

方法：我们访问了印度尼西亚城市、郊区和农村地区的成年结核病和多耐药性结核病患者，他们已至少治疗一个月，或在一个月前完成治疗。根据 WHO 的建议，我们评估了结核病造成的灾难性总成本的发生率。还分析了与发生率相关的几个阈值的敏感性，并衡量了贫困和非贫困家庭在灾难性成本发生率方面的差异。采用广义线性混合模型分析确定灾难性总成本的决定因素。

结果：共分析 282 例结核病病例和 64 例多耐药性结核病病例。结核病病例的家庭相关服务总成本中位数(四分位范围)为 133 美元(55-576)，多药耐结核病病例的相关服务费用为 2804 美元(1008-4325)。受结核病和多耐药结核病病例影响的家庭中，灾难性总支出的发生率分别为 36%(贫困家庭为 43%，非贫困家庭为 25%)和 83%(贫困和非贫困家庭均为 83%)。在受结核病影响的家庭中，灾难性总成本的决定因素是贫困家庭[校正比值比(a*OR*)=3.7，95%置信区间（*CI*）：1.7-7.8]、维持家庭生计的人(a*OR*=2.9，95% *CI*：1.3-6.6)、失业(a*OR*=21.2，95% *CI*：8.3-53.9)和以前的结核病治疗(a*OR* = 2.9，95% *CI*：1.4-6.1)。在多耐药结核病患者家庭中，确诊前有工作收入是灾难性总成本的唯一决定因素(a*OR*=8.7，95% *CI*：1.8-41.7)。

结论：尽管正在实施 UHC，受结核病影响的家庭仍然面临着灾难性总成本和随之而来的贫困。除了确保医疗保健服务的可及性外，还应提供一项降低成本的政策和额外的财政保障，以保护贫困人群和减少收入损失。

Translated from English version into Chinese by Xin-Yu Feng, edited by Pin Yang

CHINESE CENTER FOR DISEASE CONTROL AND PREVENTION
NATIONAL INSTITUTE OF PARASITIC DISEASES

Coûts totaux catastrophiques dans les foyers touchés par la tuberculose, et leurs déterminants depuis la mise en place de la couverture santé universelle en Indonésie.

Ahmad Fuady, Tanja A.J. Houweling, Muchtaruddin Mansyur, Jan Hendrik Richardus

Résumé

Rappel des faits: outre le fait d’imposer un fardeau économique aux familles touchées par la maladie, les coûts élevés associés à la tuberculose (TB) peuvent créer des obstacles à l’accès et à l’adhérence. Cela met en évidence l’urgence considérable pour atteindre l'un des objectifs de la « Stratégie pour mettre fin à la tuberculose » : aucun foyer touché par la TB ne devra faire face aux coûts catastrophiques d’ici à 2020. En Indonésie, comme ailleurs, le besoin se fait sentir également de fournir une protection sociale en mettant en place la couverture santé universelle (CSU). Nous avons dès lors évalué l'incidence des coûts totaux catastrophiques engendrés par la TB et leurs déterminants, depuis la mise en place de la CSU.

Méthodes: nous avons interviewé des patients adultes touchés par la TB et la TB multirésistante (tuberculose-MR) dans des zones urbaines, suburbaines et rurales de l'Indonésie, qui avaient été traités pendant au moins un mois, ou qui avaient terminé leur traitement depuis un mois tout au plus. D'après les recommandations formulées par l'OMS, nous avons évalué l'incidence des coûts totaux

catastrophiques engendrés par la TB. Nous avons également analysé la sensibilité de l'incidence liée à différents seuils, et mesuré les différences entre des foyers pauvres et riches en matière d'incidence de coûts catastrophiques. L'analyse de modèle mixte linéaire généralisé a permis d'identifier les déterminants des coûts totaux catastrophiques.

Résultats: nous avons analysé 282 patients touchés par la TB, et 64 patients touchés par la tuberculose -MR. Pour les services liés à la TB, la moyenne (intervalle interquartile) des coûts totaux assumés par les foyers s'élevait à 133 USD (55-576) ; pour les services liés à la tuberculose-MR, la moyenne s'élevait à 2 804 USD (1 008-4 325). L'incidence des coûts totaux catastrophiques dans tous les foyers touchés par la TB était de 36 % (43 % dans les foyers pauvres et 25 % dans les foyers riches). Pour les foyers touchés par la tuberculose-MR, l'incidence était de 83 % (83 % et 83 %). Dans les foyers touchés par la TB, les déterminants des coûts totaux catastrophiques étaient des foyers pauvres (rapport de cotes ajusté [a*RC*] = 3,7 , intervalle de confiance 95 % [*IC*]: 1,7-7,8) ; être le gagne-pain (a*RC*= 2,9 95 %*IC*: 1,3-6,6) ; perte d'emploi (a*RC*= 21,2 ; 95 %*IC*: 8,3-53,9) ; et traitement TB antérieur (a*RC*= 2,9 ; 95 %*IC*: 1,4-6,1). Dans les foyers touchés par la tuberculose-MR, être détenteur d'un emploi et percevoir un salaire avant le diagnostic était le seul déterminant des coûts totaux catastrophiques (a*RC*= 8,7 ; 95 %*IC*: 1,8-41,7).

Conclusions: Malgré la mise en place de la CSU, les foyers touchés par la TB risquent toujours de faire face à des coûts totaux catastrophiques et de s'appauvrir davantage. Outre garantir l'accès aux soins médicaux, une politique de réduction des coûts et une protection financière supplémentaire devrait être fournie pour protéger les pauvres et alléger les pertes de revenus.

Translated from English version into French by Isabelle Mathis, through

TRANSLATORS
WITHOUT BORDERS

Непосильные совокупные издержки семей, пострадавших от туберкулеза, и их детерминанты с момента внедрения Индонезией всеобщего медицинского обслуживания

Ахмад Фуади, Таня А.Ю. Хоулинг, Мухтаруддин Мансур, Ян Хендрик Ричардус

Аннотация

Справочная информация: Высокие затраты, связанные с туберкулёзом (ТБ), не только возлагают экономическое бремя на пострадавшие семьи, но и создают барьеры, препятствующие доступу и присоединению. Это подчеркивает особую важность достижения одной из целей стратегии по ликвидации ТБ: к 2020 году ни одна из страдающих от ТБ семей не должна сталкиваться с непосильными совокупными издержками. Наряду с другими странами, в Индонезии также возникает потребность в обеспечении социальной защиты посредством введения всеобщего медицинского обслуживания. По этой причине мы провели оценку охвата непосильных совокупных издержек семей, пострадавших от ТБ, а также их детерминанты с момента введения всеобщего медицинского обслуживания.

Методы: В городских, пригородных и сельских районах Индонезии мы провели опрос среди взрослых пациентов ТБ и среди больных туберкулезом с множественной лекарственной устойчивостью (МЛУ-ТБ), которые либо проходили лечение в течение как минимум одного месяца, либо завершили лечение не более, чем за месяц до проведения опроса. По рекомендации ВОЗ мы провели оценку охвата непосильных совокупных издержек, связанных с ТБ. Мы также провели анализ чувствительности охвата по отношению к нескольким пороговым значениям и измерили разницу в показателе непосильных совокупных издержек по семьям, проживающим в условиях бедности, а также другим семьям. С целью определения детерминантов непосильных совокупных издержек был проведён обобщенный линейный анализ смешанной модели.

Результаты: Мы провели анализ среди 282 пациентов с ТБ и 64 больных МЛУ-ТБ. Среднестатистические совокупные издержки семей (в межквартальном диапазоне) по медицинскому обслуживанию пациентов с ТБ составили 133 долларов США (55-576); тогда как медицинское обслуживание, связанное с МЛУ-ТБ, составило 2 804 долларов США (1 008-4 325). Охват непросильных совокупных издержек по всем семьям, страдающим от ТБ составил 36% (43% в семьях, проживающих в условиях крайней бедности и 25% в других семьях). В случае с семьями, пострадавшими от МЛУ-ТБ, охват составил 83% (83% и 83%). В семьях, пострадавших от ТБ, детерминантами непосильных совокупных издержек стали семьи, проживающие в условиях бедности (скорректированные на потенциальное соотношение шансов [a*OR*] = 3,7; 95% доверительный интервал [*CI*]: 1,7-7,8); в случае кормильца семьи (a*OR*= 2,9;95%*CI*: 1,3-6,6); потерю работы (a*OR*= 21,2; 95%*CI*: 8,3-53Ю9); а также предыдущее лечение ТБ (a*OR*= 2,9; 95%*CI*: 1,4-6,1). В случае семей, пострадавших от МЛУ-ТБ, наличие доходной работы до установления диагноза стало единственным детерминантом непосильных совокупных издержек (a*OR*= 8,7; 95%*CI*: 1,8-41,7).

Выводы: Несмотря на введение всеобщего медицинского обслуживания, семьи, пострадавшие от ТБ, до сих пор подвержены риску непосильных совокупных издержек и дальнейшего обнищания. Наряду с обеспечением гарантированного доступа к медицинскому обслуживанию, необходимо провести политику смягчения издержек и предоставить дополнительную финансовую защиту с целью оградить бедных и уменьшить потерю доходов.

Translated from English version into Russian by Liudmila Tomanek (nee Volynets), through

Costos totales catastróficos en las familias afectadas por la tuberculosis y sus determinantes desde la implementación de la cobertura sanitaria universal en Indonesia

Ahmad Fuady, Tanja A.J. Houweling, Muchtaruddin Mansyur, Jan Hendrik Richardus

Resumen

Antecedentes: aparte de suponer una carga económica para las familias afectadas, los altos costos relacionados con la tuberculosis (TB) pueden crear barreras de acceso y adherencia. Esto resalta la particular urgencia de lograr uno de los objetivos de la Estrategia Fin a la TB: que ninguna de las familias afectadas por la TB tengan que enfrentar costos catastróficos para el 2020. En Indonesia, como en otras partes, existe también una necesidad emergente de ofrecer protección social mediante la implementación de la cobertura sanitaria universal (CSU). Por lo tanto, evaluamos la incidencia de costos totales catastróficos debido a la TB y sus determinantes desde la implementación de la CSU.

Métodos: entrevistamos a pacientes adultos con TB y pacientes TB-MDR (multidrogorresistentes) en áreas urbanas, suburbanas y rurales de Indonesia, quienes habían sido tratados durante por lo menos un mes o que habían terminado el tratamiento no más de un mes antes. Siguiendo la recomendación de la OMS, evaluamos la incidencia de costos totales catastróficos debido a la TB. También analizamos la sensibilidad de la incidencia en relación con varios umbrales y medimos las diferencias entre hogares pobres y no pobres en la incidencia de costos catastróficos. Se utilizó un análisis de modelo mixto lineal generalizado para identificar los determinantes de los costos totales catastróficos.

Resultados: Analizamos 282 pacientes con TB y 64 TB-MDR. Para los servicios relacionados con la TB, la media (rango intercuartil) de los costos totales incurridos por los hogares fue de 133 dólares (55-576); para los servicios relacionados con la TB-MDR, fue de 2 804 dólares (1 008-4 325). La incidencia de costos totales catastróficos en todos los hogares afectados por la TB fue del 36% (43% en los hogares pobres y 25% en los hogares no pobres). Para las familias afectadas por TB-MDR, la incidencia fue del 83% (83% y 83%). En las familias afectadas por la TB, los determinantes de los costos totales catastróficos fueron los hogares pobres (odds ratio ajustado [*OR*a] = 3,7, intervalo de confianza [*CI*] del 95%: 1,7-7,8); ser sostén de la familia (*OR*a= 2,9,95%*CI*: 1,3-6,6); pérdida de trabajo (*OR*a= 21,2; 95%*CI*: 8,3-53,9); y tratamiento para la tuberculosis previo (*OR*a= 2,9; 95%*CI*: 1,4-6,1). En las familias afectadas por la TB-MDR, tener un trabajo remunerado antes del diagnóstico era el determinante único de costos totales catastróficos (*OR*a= 8,7; 95%*CI*: 1,8-41,7).

Conclusiones: A pesar de la implementación de la CSU, las familias afectadas por la TB todavía se arriesgan al riesgo de costos totales catastróficos y al empobrecimiento continuo. Así como garantizar el acceso a la asistencia sanitaria, es necesario proporcionar una política de mitigación del costo y protección financiera adicional para proteger a los pobres y aliviar las pérdidas de ingresos.

Translated from English version into Spanish by Rocío Tempone, through

TRANSLATORS WITHOUT BORDERS
