# Supplementary material for: Catastrophic total costs in tuberculosis-affected households and their determinants since Indonesia’s implementation of universal health coverage
Source: Infect Dis Poverty. 2018 Jan 12;7:3. doi: 10.1186/s40249-017-0382-3 (PMC5765643; doi:10.1186/s40249-017-0382-3)
Supplement: Supplementary file 2 — Reasons for not choosing public facilities at the first contact. (DOCX 15 kb) [file 40249_2017_382_MOESM2_ESM.docx]

**Additional file 2:** Reasons for not choosing public facilities at the first contact

| **Reason** | **Urban (%)** | | **Sub-urban (%)** | | **Rural (%)** | | **Total (%)** | |
| --- | --- | --- | --- | --- | --- | --- | --- | --- |
| Distance to facility | 7 | (21) | 10 | (25) | 15 | (30) | 32 | (26) |
| Accustomed to private facility | 4 | (12) | 7 | (18) | 14 | (28) | 25 | (20) |
| Mistrust towards public facility | 4 | (12) | 5 | (13) | 4 | (8) | 13 | (11) |
| No public facility available | 2 | (6) | 4 | (10) | 5 | (10) | 11 | (9) |
| Long waiting time | 4 | (12) | 2 | (5) | 1 | (2) | 7 | (6) |
| Did not know PHC offered free TB service | 1 | (3) | 1 | (3) | 5 | (10) | 7 | (6) |
| Assumed patient had common cough, not TB | 3 | (9) | 0 | (0) | 2 | (4) | 5 | (4) |
| No answer | 3 | (9) | 2 | (5) | 0 | (0) | 5 | (4) |
| PHC had limited working hours | 1 | (3) | 1 | (3) | 1 | (2) | 3 | (2) |
| Others | 4 | (12) | 8 | (20) | 3 | (6) | 15 | (12) |
